# A new species of *Liphistius* from Myanmar and description of the actual male of *L.birmanicus* Thorell, 1897 (Araneae, Mesothelae, Liphistiidae)

**DOI:** 10.3897/zookeys.1031.59102

**Published:** 2021-04-14

**Authors:** Xin Xu, Li Yu, Khin Pyae Pyae Aung, Long Yu, Fengxiang Liu, Wai Wai Lwin, Men Zing Sang, Daiqin Li

**Affiliations:** 1 College of Life Sciences, Hunan Normal University, 36 Lushan Road, Changsha 410081, Hunan Province, China; 2 State Key Laboratory of Biocatalysis and Enzyme Engineering, and Centre for Behavioural Ecology and Evolution (CBEE), School of Life Sciences, Hubei University, 368 Youyi Road, Wuhan 430062, Hubei Province, China; 3 School of Life and Environmental Sciences, University of Sydney, Sydney, NSW, Australia; 4 Department of Zoology, University of Yangon, Kamayut Township, Pyay Road, Yangon, 11041, Myanmar; 5 Department of Biology, Taungoo Education College, Taungoo, 08101, Myanmar; 6 Department of Biological Sciences, National University of Singapore, 14 Science Drive 4, 117543, Singapore

**Keywords:** Morphology, South-east Asia, taxonomy, trapdoor spiders

## Abstract

Five *Liphistius* Schiödte, 1849 species of the primitively segmented spider family Liphistiidae are currently known from Myanmar. Here, we described a new species, *Liphistiuspyinoolwin***sp. nov.** (♂♀), which was collected from Pyin Oo Lwin, Mandalay Region, Myanmar, diagnosed based on its genital morphology. The specimens (2♂♂, 5♀♀) collected by Walter C. Sedgwick from Pyin Oo Lwin in 1982 were misidentified as *L.birmanicus* Thorell, 1897 and are treated here as the newly described species. Accordingly, we described the males of *L.birmanicus* for the first time, redescribed its female, using newly collected specimens from Yadò, Than Taung and Kalekho Atet townships, Kayin State. We also provided information on the variation in genital morphology of both species, and their relationships within the *birmanicus*-group of species.

## Introduction

With its unique morphology, lifestyle (living in underground burrows), and often being regarded as ‘living fossils’ (Bristowe 1975), the primitively segmented spiders of the family Liphistiidae has fascinated many naturalists and arachnologists for over one and a half centuries, since the first species was discovered by Schiödte (1849). Recently, interest in this lineage has resurged because of its pivotal position in fully understanding the arachnid tree of life (Platnick and Gertsch 1976), and application of molecular data (Xu et al. 2015a). As the sister lineage to all other extant spiders, liphistiids bear many plesiomorphic characters, such as the presence of abdominal tergal plates and the position of the spinnerets on the median area of the ventral opisthosoma (Platnick and Gertsch 1976; Coddington and Levi 1991; Haupt 2003). In this study, we focus on the *Liphistius* Schiödte, 1849 from Myanmar, an extremely important yet very poorly studied region, because it is the westernmost distribution of liphistiids according to the current records (Thorell 1897; Platnick and Sedgwick 1984; Schwendinger 1990; Aung et al. 2019; Ono and Aung 2020) and because younger mesothele fossils have also been found in the Middle Cretaceous amber forest in northern Myanmar (Wunderlich 2017, 2019).

**Figure 1.**
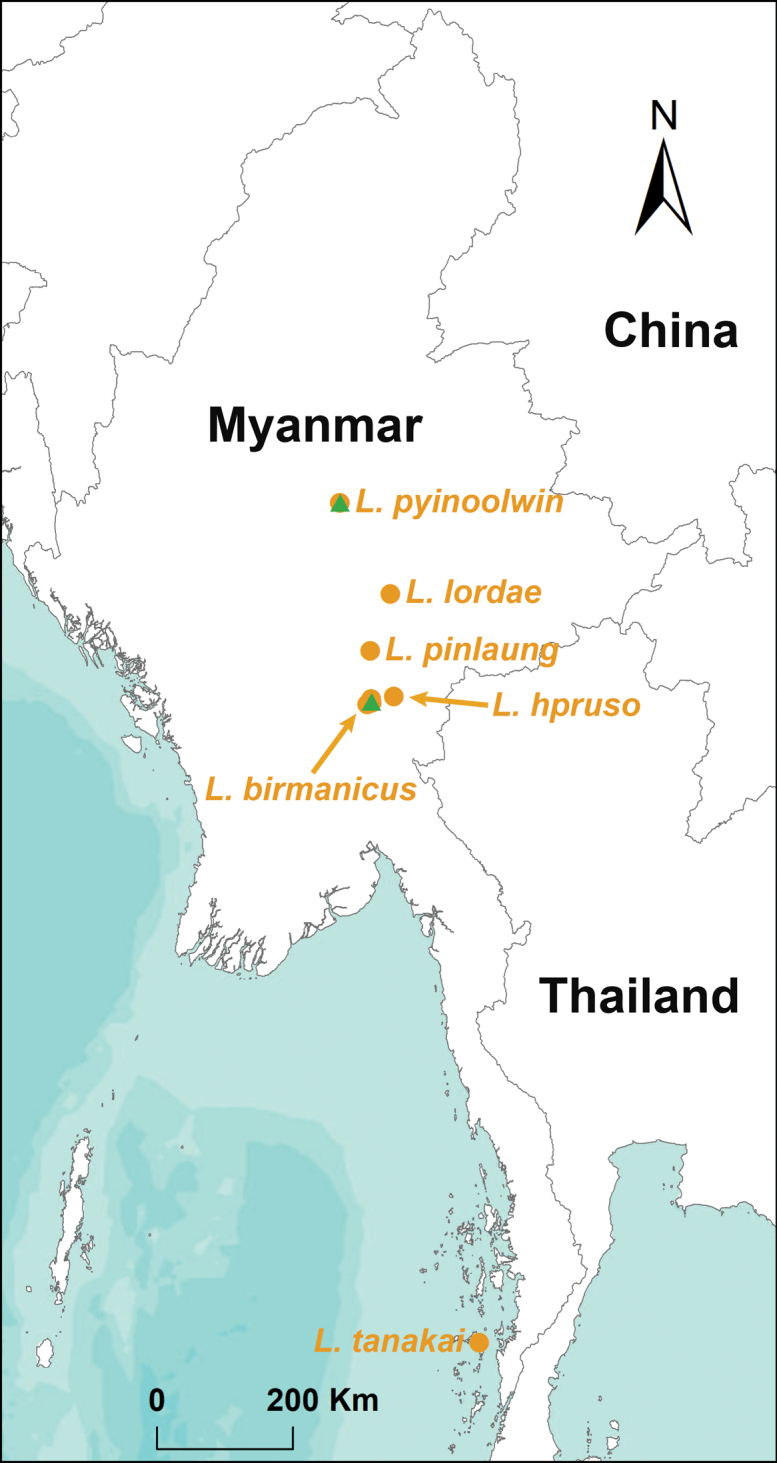
Map showing the localities of six *Liphistius* species in Myanmar including the two species described in this study. The green triangles denote all the recorded sites of adult *L.birmanicus* specimens from the literature, including the misidentified ones.

The genus *Liphistius* contains 57 nominal species and is limited to Southeast Asia (Indonesia (Sumatra), Laos, Malaysia, Myanmar, and Thailand) (Xu et al. 2015b; World Spider Catalog 2021). Out of 57 species, 32 *Liphistius* species have been reported from Thailand (World Spider Catalog 2021). Given that Myanmar and Thailand share similar landmass, climate and geological topography, a comparable species diversity is expected for Myanmar. However, only five species (*L.birmanicus* Thorell, 1897, *L.hpruso*Aung et al., 2019, *L.lordae* Platnick & Sedgwick, 1984, *L.pinlaung*Aung et al., 2019, and *L.tanakai* Ono & Aung, 2020) have been described from Myanmar so far (Fig. 1). This is probably due to the lack of local arachnologists and the difficulty of accessibility to foreign arachnologists. Working on Myanmar *Liphistius* is thus vital to fully understanding the geographic distribution and species diversity of liphistiids.

In spite of only five described species, the taxonomy of Myanmar *Liphistius*, including *L.birmanicus*, seems to be problematic. The female type of *L.birmanicus*, which was designed as the lectotype by Platnick and Sedgwick (1984), was collected from Yadò Village (Kayin State since 1989, formerly known as Kayah or Karen State), by an Italian explorer, Leonardo Fea, during his expedition to Myanmar between 1885 and 1889, and described by Thorell in 1897. Another specimen, a damaged immature male, recorded by Gravely (1915) from Mawlamyine (formerly Moulmein, Mon State), far south from Yadò, was considered as *L.birmanicus* (Bristowe 1938), but is still unclear (Schwendinger 1990). In 1984, *L.birmanicus* was redescribed based on the specimens collected from Pyin Oo Lwin (formerly Maymyo, Mandalay Region) instead from the type locality Yadò (Platnick and Sedgwick 1984). However, we believe that the specimens from Pyin Oo Lwin might not be *L.birmanicus*. The issue with *L.birmanicus* should thus be addressed before further studying Myanmar *Liphistius* species.

To resolve the *L.birmanicus* issue, and to document *Liphistius* species diversity, which could allow exploring how geological and climatic events may have shaped its biogeographical history and its diversity in Myanmar, we undertook three expeditions to Myanmar in 2018 and 2019. In this study, we describe *L.birmanicus* males for the first time and redescribe the females based on the female lectotype and newly collected specimens from the type locality. We also diagnose and describe the specimens collected from Pyin Oo Lwin, misidentified as *L.birmanicus* by Platnick and Sedgwick (1984) and Schwendinger (1990), as a new species.

## Materials and methods

### Specimen collection

**Figure 2.**
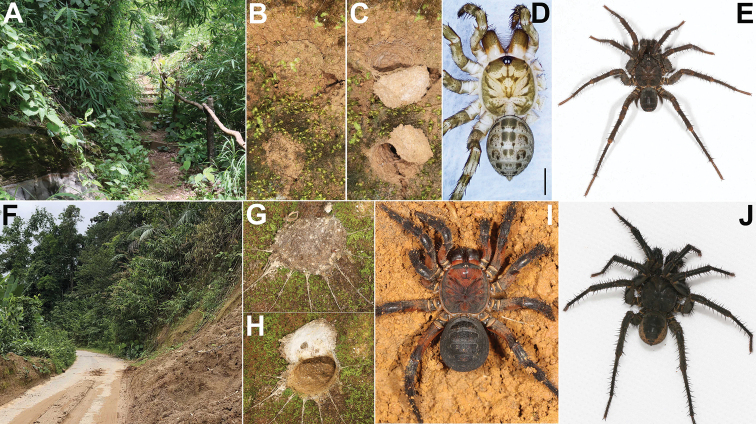
Microhabitats, burrows, and general somatic morphology of *Liphistiuspyinoolwin* sp. nov. and *Liphistiusbirmanicus* Thorell, 1897 **A–E***L.pyinoolwin* sp. nov. **A** microhabitat **B** a burrow with two trapdoors closed **C** same, trapdoors opened **D** female (XUX–2018–094) **E** male (XUX–2018–110B) **F–J***L.birmanicus***F** microhabitat **G** burrow with trapdoor closed **H** same, trapdoor opened **I** female (ARAMYN–090) **J** male (ARAMYN–096); Scale bar: 2 mm (**D**).

All specimens were collected from Pyin Oo Lwin (Mandalay Region), Than Taung and Kalekho Atet townships (Kayin State), Myanmar (Figs 1, 2). They were captured alive and fixed in absolute ethanol. Their right four legs were then removed, preserved in absolute ethanol, and stored at −80 °C for molecular work. The remains of each specimen were preserved in 80% ethanol as vouchers for morphological examination. All type and voucher specimens were deposited at the Centre for Behavioural Ecology and Evolution (**CBEE**), College of Life Sciences, Hubei University, Wuhan, Hubei Province, China.

### Morphological examination

Specimens were examined under an Olympic SZX16 stereomicroscope. Female genitalia were cleared using 10 mg/ml trypsase (Bomei Biotech Company, Hefei, Anhui, China) for at least three hours in room temperature to dissolve soft tissues, examine, and photograph with a digital camera CCD mounted on an Olympic BX53 compound microscope. Genital anatomical terminology follows Schwendinger et al. (2019) and Aung et al. (2019). All measurements were carried out under a Leica M205 digital microscope using the software of Leica Application Suite v4 and are given in millimetres. Leg and palp measurements are given in the following order: total leg length (femur + patella + tibia + metatarsus + tarsus), total palp length (femur + patella + tibia + tarsus). Abbreviations used in the text are: **ALE** = anterior lateral eye; **AME** = anterior median eye; **CDO** = central dorsal opening; **CT** = contrategulum; **E** = embolus; **GA** = genital atrium; **PC** = paracymbium; **PeP** = paraembolic plate; **PLE** = posterior lateral eye; **PME** = posterior median eye; **PPl** = poreplate; **PS** = posterior stalk; **RC** = receptacular cluster; **ST** = subtegulum; **T** = tegulum; **TiA** = tibial apophysis. **AMNH** = American Museum of Natural History, New York; **IZC** = Invertebrate Zoology Code at AMNH; **MCSNG** = Museo Civico di Storia Naturale, Genova, Italy; **MCZ** = Museum of Comparative Zoology, Harvard University, Cambridge, MA.

## Taxonomy

### Family Liphistiidae Thorell, 1869

#### Subfamily Liphistiinae Thorell, 1869

##### Genus Liphistius

Taxon classificationAnimaliaAraneaeLiphistiidae

Schiödte, 1849

C40D04C0-11E7-5959-9C72-319816399A06

###### Type species.

*Liphistiusdesultor* Schiödte, 1849.

###### Diagnosis.

**Figure 3.**
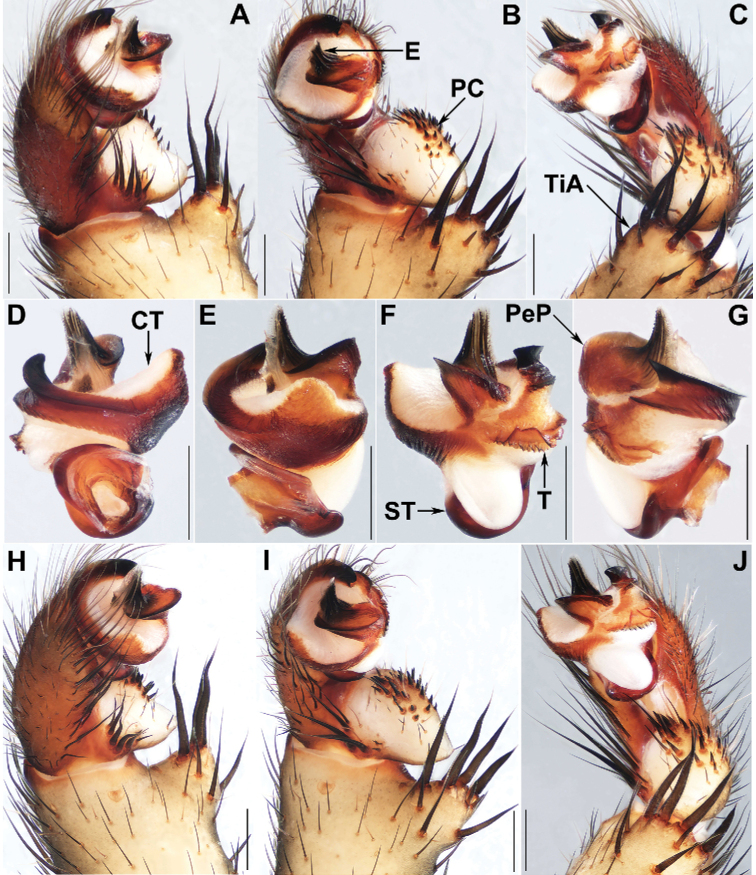
Male genital anatomy of *Liphistiuspyinoolwin* sp. nov. **A, H** palp prolateral view **B, I** palp ventral view **C, J** palp retrolateral view **D–G** palp distal view **A–C** XUX–2018–089 **D–G** XUX–2018–110B **H–J** XUX–2018–098; Scale bars: 0.5 mm.

**Figure 4.**
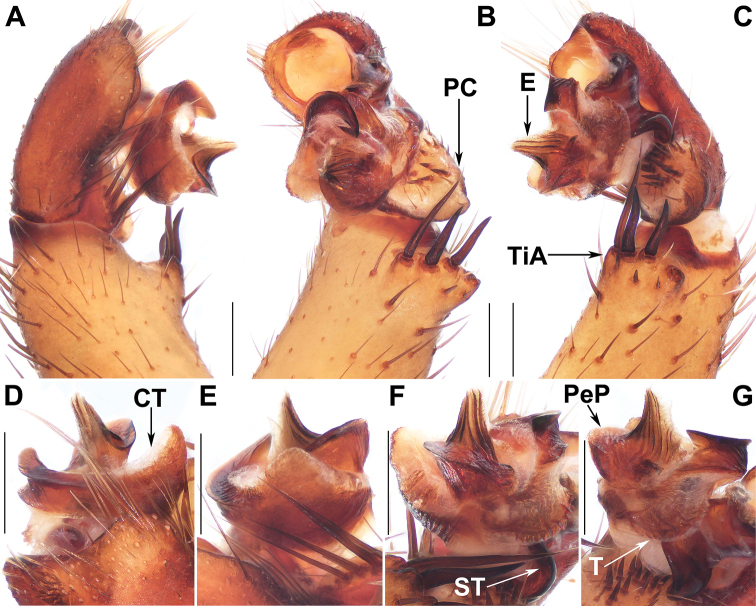
Male genital anatomy of *Liphistiuspyinoolwin* sp. nov. (specimens from AMNH, collected by W. Sedgwick) **A** palp prolateral view **B** palp ventral view **C** palp retrolateral view **D–G** palp distal view **A–G** AMNH_IZC 00356855; Scale bars: 0.5 mm.

**Figure 5.**
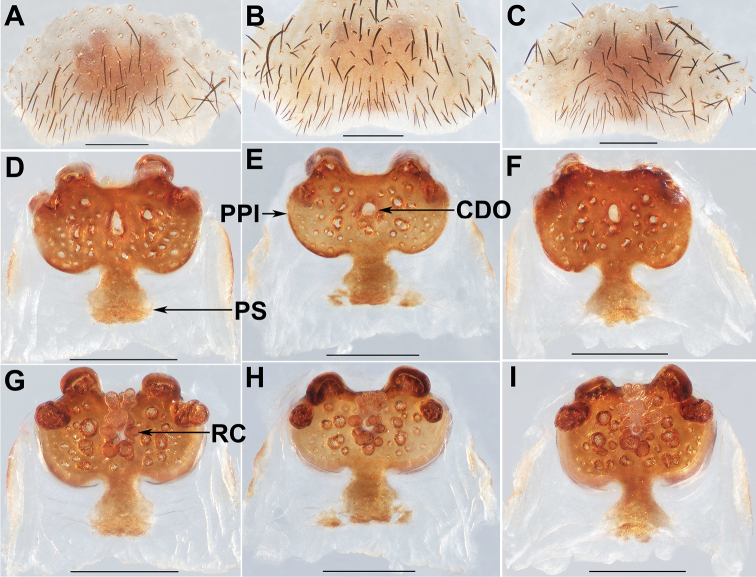
Female genital anatomy of *Liphistiuspyinoolwin* sp. nov. **A–C** plate ventral view **D–F** vulva dorsal view **G–I** vulva ventral view **A, D, G** XUX–2018–094 **B, E, H** XUX–2018–096 **C, F, I** XUX–2018–104; Scale bars: 0.5 mm.

**Figure 6.**
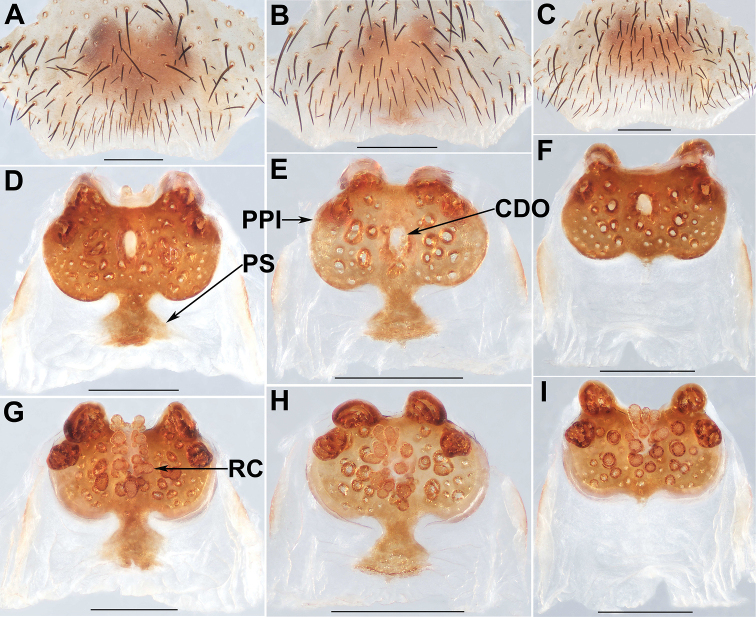
Female genital anatomy of *Liphistiuspyinoolwin* sp. nov. **A–C** plate ventral view **D–F** vulva dorsal view **G–I** vulva ventral view **A, D, G** XUX–2018–105 **B, E, H** XUX–2018–109 **C, F, I** XUX–2018–110; Scale bars: 0.5 mm.

**Figure 7.**
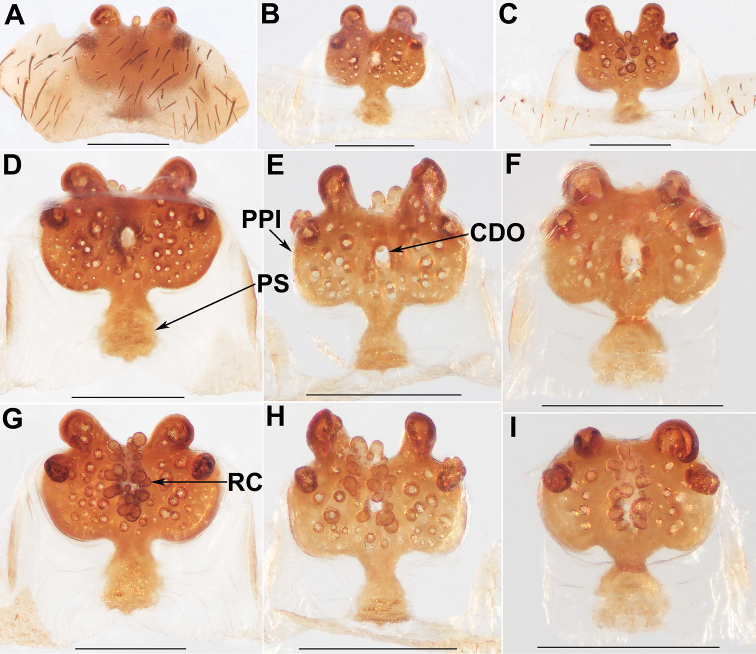
Female genital anatomy of *Liphistiuspyinoolwin* sp. nov. (specimens from AMNH, collected by W. Sedgwick) **A** plate ventral view **B, D–F** vulva dorsal view **C, G–I** vulva ventral view **A, D, G** AMNH_IZC 00356859 **B, C** AMNH_IZC 00356856 **E, H** AMNH_IZC 00356857 **F, I** AMNH_IZC 00356858; Scale bars: 0.5 mm.

*Liphistius* differs from all other liphistiid genera by the presence of a tibial apophysis on male palp (Figs 3A–C, 3H–J, 4A–C 8A–C), and by the presence of a poreplate and a median receptacular cluster in female genitalia (Figs 5D–I, 6D–I, 7B–I, 9D–I, 10C–G).

###### Distribution.

Indonesia (Sumatra), Laos, Malaysia, Myanmar, and Thailand.

##### Liphistius pyinoolwin sp. nov.

Taxon classificationAnimaliaAraneaeLiphistiidae

DE82617D-DC96-55A9-A0F6-960C0993BDDD

http://zoobank.org/781BD6EE-9EC1-4C53-944A-9F45E2F44AE9

Figs 2 , 3 , 4 , 5 , 6 , 7

Liphistius birmanicus Platnick & Sedgwick, 1984: 8 (only 2♂♂ 5♀♀ from Pyin Oo Lwin, Mandalay Region, Myanmar, alt. 1150 m; collected by W. Sedgwick on 13 July 1982; deposited in AMNH (2♂♂ 4♀♀; examined) and MCZ (1♀; not examined)), misidentification, partim; Schwendinger, 1990: 331–332 (illustration based on 2♂♂ 4♀♀ (AMNH)), misidentification.

###### Type material.

***Holotype***: Mynamar · ♂; Mandalay Region, Pyin Oo Lwin District, Pyin Oo Lwin township, Anesakhan Village, near Dat Taw Gyaint Waterfall Resort, the View Resort & Restaurant; 21.98°N, 96.38°E; alt. 908 m; 13 July 2018; D. Li, F.X. Liu, X. Xu and L. Yu leg.; XUX–2018–089. ***Paratypes***: Myanmar · 7 ♂♂, 15 ♀♀; same data as for the holotype; XUX–2018–090, 093, 094, 096, 098, 099A, 102, 103, 103A, 104, 104A, 105, 106, 107–110, 110A, 110B, 110C, 111, 111A.

***Other material***: Myanmar · 1 ♂, 4 ♀♀ (AMNH; examined); Mandalay, Pyin Oo Lwin; alt. 1150 m; 13 July 1982; W. Sedgwick leg.; AMNH_IZC 00356855 (♂; matured on 14 October 1982, died on 23 February 1983), AMNH_IZC 00356856 (♀; moulted on 28 February 1983, died on 17 April 1983), AMNH_IZC 00356857 (♀; moulted on 27 January 1983, died on 14 February 1983), AMNH_IZC 00356858 (♀; died on 15 October 1982), AMNH_IZC 00356859 (♀; moulted on 6 November 1982, died on 1 March 1983).

###### Diagnosis.

Males of *L.pyinoolwin* sp. nov. can be distinguished from those of *L.birmanicus*, *L.lahu* Schwendinger, 1998, *L.lordae*, and *L.pinlaung* by the presence of a lateral process on the paracymbium (Figs 3A, H, I, 4B); from those of *L.birmanicus* by the larger tibial apophysis (Fig. 3A, B, H, I), the plane cumulus (Figs 3A, B, H–J, 4A), the smaller paraembolic plate (Figs 3A–J, 4F, G), and the wider shorter contrategular process (Figs 3E, 4F); from those of *L.lahu* by the narrower tegulum (Figs 3C, F, J, 4F, G); from those of *L.lordae* by the wider tibial apophysis at base (Figs 3A, B, H, I, 4B), and the shorter, less regularly arranged setae on the cumulus (Figs 3A, B, H, I, 4A); from those of *L.pinlaung* by the tegulum with a slightly dentated margin (Figs 3C, F, J, 4F). Females of *L.pyinoolwin* sp. nov. resemble those of *L.birmanicus*, *L.hpruso* and *L.pinlaung* by the poreplate with two pairs of lobes, but can be distinguished from those of *L.birmanicus* and *L.pinlaung* by the small, narrower posterior stalk (Figs 5D–I, 6D, E, G, H, 7B–I), as well as the narrower, longer receptacular cluster (Figs 5G–I, 6G–I, 7C, G–I); from those of *L.hpruso* by the poreplate with larger anterior lobes (Figs 5D–I, 6D–I, 7A–I); from those of the other *Liphistius* by the poreplate with four anterior lobes (Figs 5G–I, 6G–H, 7A–I).

###### Description.

***Male*** (holotype). Total length, excluding chelicerae, 13.95. Carapace 6.03 long and 6.17 wide, black brown, furnished with a few short, scattered bristles. ALE>PLE>PME>AME, eye sizes and interdistances: AME 0.10, ALE 0.60, PME 0.21, PLE 0.42, AME–AME 0.07, AME–ALE 0.09, PME–PME 0.11, PME–PLE 0.08, ALE–PLE 0.09, ALE–ALE 0.06, PLE–PLE 0.36, AME–PME 0.09. Chelicerae robust, promargin of chelicerae groove with 12 strong denticles of variable size. Labium 0.61 long and 1.09 wide, wider than long, fused with sternum. Sternum 2.77 long and 1.03 wide, longer than wide, and a few weakly spined setae on the anterior tip and many long spined setae on the posterior tip, elongated posterior tip. Opisthosoma 6.71 long and 5.00 wide, black, with 12 tergites, the fifth largest, 8 spinnerets. Legs without distinct annulations. Superior tarsal claws of anterior legs with 3 or 4 teeth, of posteriors with 4. Measurements: leg I 15.73 (4.42 + 1.39 + 3.78 + 3.80 + 2.34), leg II 16.16 (4.31 + 1.36 + 3.49 + 4.38 + 2.62), leg III 18.09 (4.15 + 1.34 + 4.16 + 5.68 + 2.76), leg IV 23.69 (5.66 + 1.57 + 5.18 + 7.66 + 3.62).

Palp: Tibial apophysis very broad at base, with four long, stouter setae with similar lengths and a few short spines (Figs 3A–C, H–J, 4A–C); paracymbium wide, with pointed lateral process and many setae situated at the tip, and several tapering spines on the plane cumulus (Figs 3A, B, H, I, 4A); subtegular apophysis well developed (Figs 3C, F, J, 4F, G); contrategulum with a conical, short, blunt-tipped process (Figs 3D–F, 4E), distal edge widely arched, with a smooth sharp projection (Figs 3B, D, F, G, 4F, G); tegulum small, with a slightly dentated margin (Figs 3C, F, J, 4F, G); paraembolic plate short, widely rounded (Figs 3A–J, 4E–G); embolus short conical, basally sclerotized, with 6 longitudinal ridges that reach the tip, embolic parts adjacent (Figs 3A–J, 4C–G).

***Female*** (XUX–2018–094, Fig. 2D). Total length, excluding chelicerae, 10.40. Carapace 4.79 long, 4.72 wide, light brown, furnished with few short, scattered bristles (Fig. 2D). Eight eyes on darkened ocular tubercle, ALE > PLE > PME > AME. Eye sizes and interdistances: AME 0.06, ALE 0.45, PME 0.19, PLE 0.35; AME–AME 0.08, AME–ALE 0.10, PME–PME 0.05, PME–PLE 0.10, ALE–PLE 0.05, ALE–ALE 0.10, PLE–PLE 0.33, AME–PME 0.06. Chelicerae light and glabrous proximally, robust, dark brown; promargin of chelicerae groove with 11–12 denticles of variable size. Labium 0.59 long, 1.25 wide. Sternum 2.55 long, 1.23 wide, light brown with several setae. Opisthosoma 5.44 long, 3.84 wide, brown, with 12 tergites, and 8 spinnerets. Legs brown with strong hairs and spines, long and short black sparse setae, with three tarsal claws. Measurements: palp 7.89 (2.84 + 1.05 + 1.98 + 2.02), leg I 10.16 (3.33 + 1.32 + 2.22 + 1.90 + 1.39), leg II 9.95 (3.15 + 1.07 + 2.14 + 2.03 + 1.56), leg III 11.10 (3.18 + 1.19 + 2.55 + 2.58 + 1.60), leg IV 15.60 (4.41 + 1.24 + 3.53 + 3.91 + 2.51).

Female genitalia: Posterior margin of genital sternite curved (Figs 5A–C, 6A–C, 7A); approximately rectangular poreplate wider than long, with a pair of large, well separated anterior lobes and a pair of small anterolateral lobes; the anterior lobes very close to the anterolateral lobes (Figs 5D–I, 6D–I, 7B–I); transition between poreplate and posterior stalk distinct (Figs 5D–I, 6D, E, G, H, 7B–I); posterior stalk long, narrow; racemose receptacular cluster long and narrow, central dorsal opening sphere-shaped (Figs 5D–F, 6D–F, 7D–F).

###### Etymology.

The species epithet “pyinoolwin” is a toponym referring to the type locality.

###### Distribution.

Myanmar (Mandalay Region).

###### Variation.

Body size: males (*N*=8): BL 8.63–13.95, CL 4.23–6.03, CW 4.87–6.17, OL 3.76–6.71, OW 2.85–5.17; females (*N*=15): BL 10.40–14.21, CL 4.79–6.37, CW 4.55–5.91, OL 5.44–8.10, OW 3.84–6.27; The examined females have different genitalia, including the specimen XUX–2018–110, which lack the posterior stalk (Fig. 6F, 6I); the shape of the anterior and anterolateral lobes of the poreplate is variable (Figs 5G–I, 6G–I, 7C, 7G–I); In some specimens, the receptacular cluster is beyond the anterior margin of the poreplate dorsally (Figs 5D, 6D, 7A, D, E), whereas in others are not (Figs 5E, F, 6E, F, 7B, F), and the size and shape of the receptacular cluster may be slightly different (Figs 5G–I, 6G–I, C, G–I); the shape and size of the central dorsal opening are also variable (Figs 5D–F, 6D–F, 7B, D–F).

###### Remarks.

We examined 8 males and 15 females collected from Pyin Oo Lwin by us, as well as 1 male and 4 females collected by W. Sedgwick on 13 July 1982, which were used to redescribe *L.birmanicus* by Platnick and Sedgwick (1984) and reviewed by Schwendinger (1990). After examined the male and females collected by W. Sedgwick, even though the male palp was distorted (Fig. 4A–C), we can still identify it as the same as the descriptions and illustrations by Platnick and Sedgwick (1984), and the same as the males and females collected by us at Pyin Oo Lwin.

##### Liphistius birmanicus

Taxon classificationAnimaliaAraneaeLiphistiidae

Thorell, 1897

8A0D34CB-54B3-5000-B1B7-0FC20925A208

Figs 2 , 8 , 9 , 10

Liphistius birmanicus Thorell, 1897: 162 (♀, from Yadò, Kayin State, Myanmar; alt. 1200–1300 m; 1885–1889, collected by L. Fea; deposited in MCSNG, examined); Pocock, 1900: 156; Bristowe, 1933: 1029; Haupt, 1983: 280.

###### Material examined.

Mynamar · 7♀♀; Kayin State, Than Taung township, Yadò; 19.33°N, 96.81°E; alt. 1062–1090 m; ARAMYN–496, 497, 498, 501, 504, 505, 506; 2♂♂, 3♀♀; Kayin State, Kalekho Atet township; 19.31°N, 96.75°E; alt. 554–564 m; 15 November 2018; D. Li and L. Yu leg.; ARAMYN–090, 091, 092, 095, 096. ***Other material***: Mynamar · 1♀ (lectotype); Kayin State (formerly Kayah State: Platnick and Sedgwick 1984; Karen State: Schwendinger 1990), Yadò, Mt. Chebà; alt. 1200–1300 m; 1885–1889; L. Fea leg. (MCSNG; examined).

###### Diagnosis.

**Figure 8.**
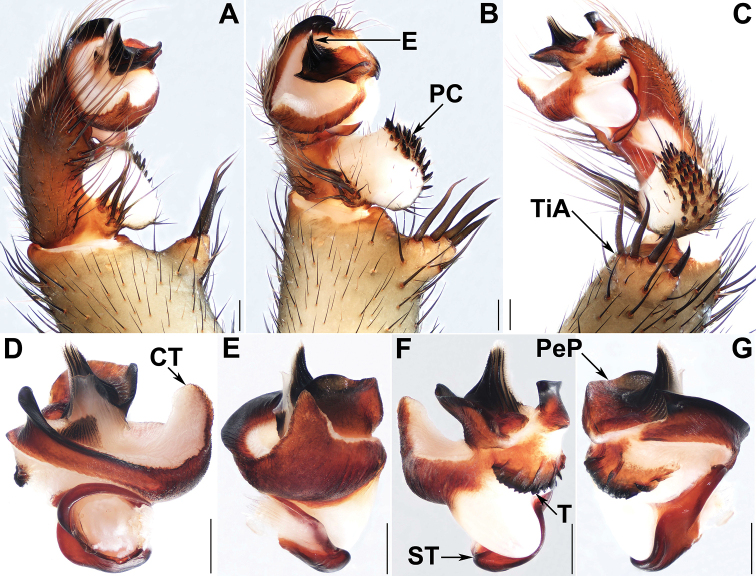
Male genital anatomy of *Liphistiusbirmanicus* (Thorell, 1897) **A** palp prolateral view **B** palp ventral view **C** palp retrolateral view **D–G** palp distal view **A–C** ARAMYN–096 **D–G** ARAMYN–092; Scale bars: 0.5 mm.

**Figure 9.**
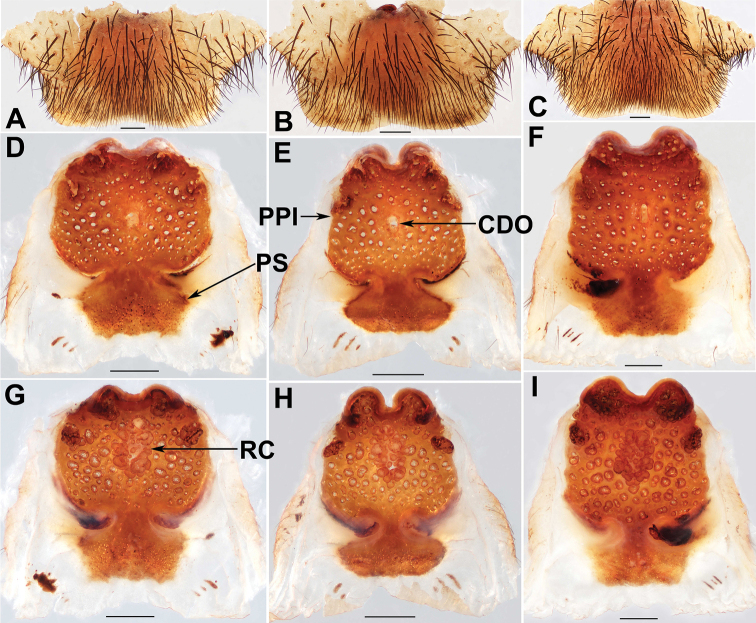
Female genital anatomy of *Liphistiusbirmanicus* (Thorell, 1897) **A–C** plate ventral view **D–F** vulva dorsal view **G–I** vulva ventral view **A, D, G** ARAMYN–497 **B, E, H** ARAMYN–501 **C, F, I** ARAMYN–506; Scale bars: 0.5 mm.

**Figure 10.**
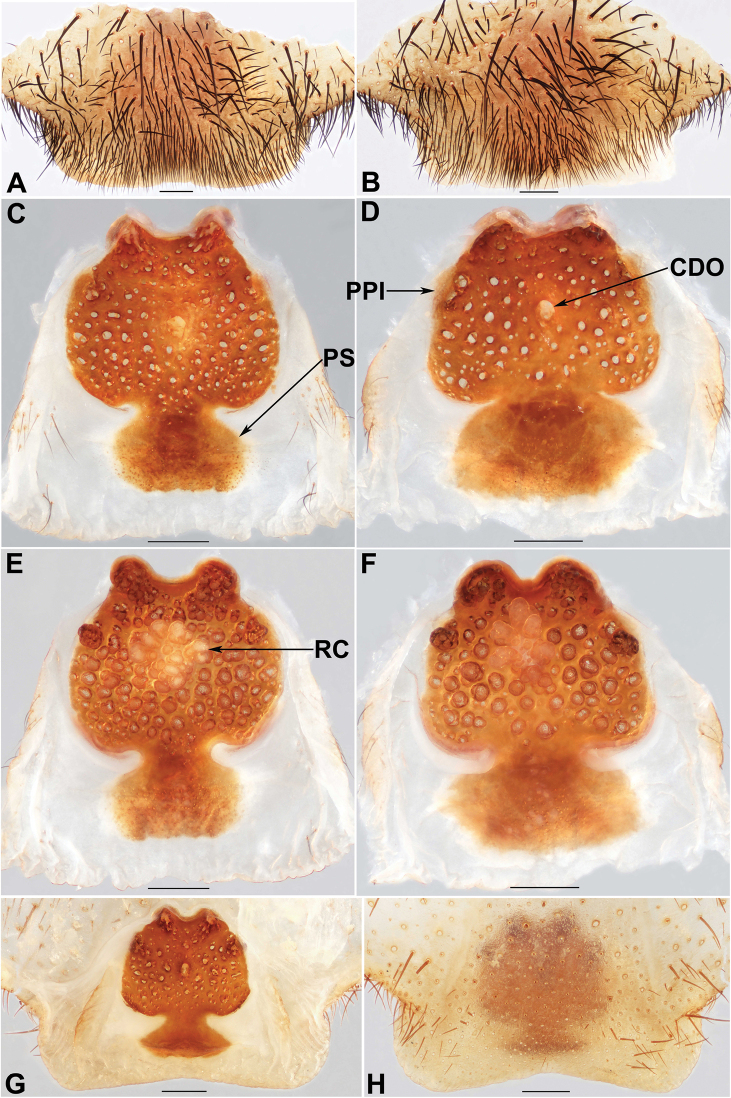
Female genital anatomy of *Liphistiusbirmanicus* (Thorell, 1897) **A, B** plate ventral view **C, D, G** vulva dorsal view **E, F, H** vulva ventral view **A, C, E** ARAMYN–091 **B, D, F** ARAMYN–095 **G, H** lectotype (specimen from MCSNG, collected by L. Fea); Scale bars: 0.5 mm.

Males of *L.birmanicus* can be distinguished from those of *L.pyinoolwin* sp. nov. by the lack of the lateral process of the paracymbium (Fig. 8A–C), the cumulus slightly raised (Fig. 8B); the wider paraembolic plate (Fig. 8B, C, F), the narrower, longer contrategular process (Fig. 8D–F), and the slightly smaller tibial apophysis (Fig. 8A–C); differ from those of *L.pinlaung* by the larger tibial apophysis (Fig. 8A–C), and by the raised cumulus with shorter setae (Fig. 8A, B); from those of *L.lahu* by the larger paraembolic plate and the cumulus with shorter setae (Fig. 8B); from those of *L.lordae* by the wider tibial apophysis at base (Fig. 8A, B), and the raised cumulus with shorter, less regularly arranged setae (Fig. 8B); Females of *L.birmanicus* resemble those of *L.hpruso*, *L.pinlaung* and *L.pyinoolwin* sp. nov. by the poreplate with two pair of lobes but can be distinguished from those of *L.hpruso* and *L.pyinoolwin* sp. nov. by the broad posterior stalk and the poreplate slightly longer than wide (Figs 9D–I, 10C–G); from those of *L.pinlaung* by the broader, axe-blade-shaped posterior stalk and the smaller anterolateral lobes of the poreplate (Figs 9C–I, 10E, F); from those of the other *Liphistius* by the poreplate with four anterior lobes (Figs 9G–I, 10E–F).

###### Description.

***Male*** (ARAMYN–096, Fig. 2J). Total length, excluding chelicerae, 19.90. Carapace 9.50 long and 9.45 wide, black, furnished with few short, scattered bristles. ALE>PLE>PME>AME, eye sizes and interdistances: AME 0.14, ALE 0.91, PME 0.33, PLE 0.62, AME–AME 0.11, AME–ALE 0.16, PME–PME 0.09, PME–PLE 0.15, ALE–PLE 0.09, ALE–ALE 0.18, PLE–PLE 0.45, AME–PME 0.06. Chelicerae robust, promargin of chelicerae groove with 11 denticles of variable size. Labium 1.01 long and 1.38 wide, wider than long, fused with sternum. Sternum 4.82 long and 1.12 wide, longer than wide, and strong spined, elongated anterior and posterior tip. Opisthosoma 9.67 long and 7.39 wide, with 12 black tergites, the fifth largest, 8 spinnerets. Legs with strong hairs and spines. Measurements: leg I 16.99 (4.32 + 2.55 + 3.55 + 4.66 + 1.92), leg II 18.06 (4.32 + 2.41 + 3.74 + 5.18 + 2.41), leg III 18.46 (4.44 + 1.85 + 2.83 + 6.68 + 2.66), leg IV 20.40 (3.56 + 1.52 + 4.25 + 8.46 + 2.63).

Palp: Tibial apophysis with four long setae with different lengths (Fig. 8B, C), paracymbium large, wide, with many setae at the tip and several tapering spines on the slightly raised cumulus (Fig. 8A–C); subtegular apophysis well developed (Fig. 8C, F); contrategulum with a triangular process, distal edge widely arched, with a smooth sharp projection (Fig. 8D, E, F); tegulum small, terminal apophysis with finely dentated margin (Fig. 8C, F, G); paraembolic plate base wide with a curved margin (Fig. 8D, G); embolus long and conical, basally sclerotized, with 7 longitudinal ridges that reach the tip, embolic parts adjacent (Fig. 8D–G).

***Female*** (ARAMYN–091). Total length, excluding chelicerae, 22.50. Carapace 11.88 long and 11.06 wide, reddish black, furnished with few short, scattered bristles. Eight eyes on darkened ocular tubercle, ALE > PLE > PME > AME, eye size and interdistances: AME 0.16, ALE 0.92, PME 0.38, PLE 0.71, AME–AME 0.13, AME–ALE 0.18, PME–PME 0.15, PME–PLE 0.12, ALE–PLE 0.09, ALE–ALE 0.20, PLE–PLE 0.68, AME–PME 0.13. Chelicerae proximally glabrous, robust, reddish black; promargin of chelicerae groove with 11 strong denticles of variable size. Labium 1.40 long, 2.01 wide. Sternum 4.42 long, 1.68 wide, strong spined, elongated posterior tip. Opisthosoma 10.46 long, 8.31 wide, black, with 12 tergites, the fifth largest, and 8 spinnerets (Fig. 2I). Legs reddish black with strong hairs and spines, long and short black sparse setae, legs each with three tarsal claws. Measurements: palp 16.92 (6.17 + 2.32 + 4.82 + 3.61), leg I 23.27 (7.81 + 2.78 + 5.38 + 4.65 + 2.65), leg II 24.41 (7.85 + 2.85 + 5.57 + 5.32 + 2.82), leg III 26.88 (7.82 + 3.01 + 5.97 + 6.52 + 3.56), leg IV 35.45 (10.11 + 2.13 + 7.85 + 10.82 + 4.54).

Female genitalia: Posterior margin of genital sternite slightly curved (Figs 9A–C, 10A, H); poreplate almost squared, with a pair of large anterior lobes and a pair of small anterolateral lobes (Figs 9G–I, 10E, F); anterior and anterolateral lobes well separated (Figs 9G–I, 10E, F); indistinct transition between the poreplate and posterior stalk (Figs 9D–I, 10D); posterior stalk broad, large, constricted at base, axe-blade-shaped (Figs 9D–I, 10C–G); racemose receptacular cluster large (Figs 9G–I, 10E, F); central dorsal opening small, spheric (Figs 9D–F, 10C, D, F).

###### Distribution.

Myanmar (Than Taung and Kalekho Atet townships, Kayin State).

###### Variation.

Body size: males (*N*=2): BL 18.58–19.90, CL 9.05–9.50, CW 8.01–9.45, OL 9.08–9.67, OW 6.95–7.39; females (*N*=10): BL 14.45–25.95, CL 6.41–12.26, CW 5.45–12.71, OL 7.65–17.09, OW 6.47–14.76; in ventral view, the shape of the transition between poreplate and posterior stalk is different between the specimens ARAMYN–497, 501, 506 (Fig. 9) and ARAMYN–091, 095 (Fig. 10C–F); anterior lobes larger and close to each other (ARAMYN–501, Fig. 9E, H) compared to other specimens (Figs 9G, I, 10E, F); the size and shape of the receptacular cluster are different (Figs 9G–I, 10E, F); and the shape of central dorsal opening is also variable (Figs 9D–F, 10C, D, G).

###### Remarks.

Only 4 specimens were collected from Myanmar before 1984, all of them identified as *L.birmanicus* in the literature. One female and two juvenile specimens were collected from Yadò and Biapò by Leonardo Fea, most likely in the years of 1887–1888 (Fea 1888) during his expedition to Karen Hills or Kayah-Karen Mountains (Bolotov et al. 2019). These 3 specimens were deposited in MCSNG, Italy. The adult female used to be described as *L.birmanicus* by Thorell in 1897, then redescribed by Pocock (1990), Bristowe (1932), and illustrated by Haupt (1983). Two juvenile specimens were only mentioned in Thorell’s description (1897) and have never been mentioned since then. The fourth specimen, an immature male collected from Mawlamyine, was first mentioned by Gravely (1915), and considered as *L.birmanicus* by Bristowe (1938). However, Schwendinger (1990) questioned its status, as do we, because the geographic locality is very far from the type locality, Yadò, and it is immature. Nevertheless, new specimens from Mawlamyine are needed to resolve this issue in the future.

One specimen collected outside Myanmar was identified as *L.birmanicus*, but it is actually not a *Liphistius*. Berlard (1932: figure 443) illustrated and assigned a male to *L.birmanicus*, which was collected from the forest of Kha-16, Tonkin, in the district of Song-Luc-Nam, Vietnam. It is obviously not a *Liphistius* since it lacks a palpal tibial apophysis. Simon (1908) first identified it as *L.birmanicus*, but Bristowe (1933) described it as a distinct species, *L.tonkinensis*, presently *Vinathelatonkinensis* (Bristowe, 1933) (Xu et al. 2015a; World Spider Catalog 2021).

Platnick and Sedgwick (1984) provided illustrations and detailed descriptions of *L.birmanicus* after examining the lectotype from Yadò (deposited in MCSNG). Their descriptions of male and female were based on the specimens collected from Pyin Oo Lwin by W. Sedgwick instead of the lectotype. Schwendinger (1990) also provided illustrations and assigned those Pyin Oo Lwin specimens to *L.birmanicus*. As they had noticed, compared to Pyin Oo Lwin females, the female lectotype is much larger (Platnick and Sedgwick 1984; Schwendinger 1990), although the body size is not usually used for identifying a species. Moreover, the poreplate of the lectotype possesses relatively smaller anterior lobes and a much wider posterior stalk as illustrated in Haupt (1983). Thus, we treated the Pyin Oo Lwin specimens as a distinct species, here described as *L.pyinoolwin* sp. nov..

###### Relationships.

*Liphistiuspyinoolwin* sp. nov. belongs to the *birmanicus*-group that currently contains *L.birmanicus*, *L.hpruso*, *L.lordae*, *L.lahu*, and *L.pinlaung* based on the male and female genital morphology. Since Schwendinger (1998) provided a detailed discussion about the shared characters among the group members, we give two additional characters within the group here. The *birmanicus*-group can be divided into two types, one including *L.birmanicus*, *L.hpruso*, *L.pinlaung*, and *L.pyinoolwin* sp. nov., the other including *L.lahu* and *L.lordae*, based on the following synapomorphies: female poreplate of the former four species has four anterior lobes, while female poreplate of the latter two species has only two anterior lobes (Figs 5, 6, 7, 9, 10); the male palp of the former four species has shorter, less regularly arranged setae on the cumulus, and a wider tibial apophysis at base compared with the latter two species (Figs 3A, B, 8A, B).

## Supplementary Material

XML Treatment for Liphistius

XML Treatment for Liphistius pyinoolwin

XML Treatment for Liphistius birmanicus
